# Resilience Factors Contributing to Mental Health Among People Affected by the Fukushima Disaster: Development of Fukushima Resilience Scale

**DOI:** 10.3389/fpubh.2020.00159

**Published:** 2020-05-06

**Authors:** Yui Takebayashi, Masaharu Maeda, Masatsugu Orui, Satomi Nakajima, Maho Momoi, Akiko Ito, Hideki Sato, Seiji Yasumura, Hitoshi Ohto

**Affiliations:** ^1^Department of Disaster Psychiatry, Fukushima Medical University School of Medicine, Fukushima, Japan; ^2^Radiation Medical Science Center for the Fukushima Health Management Survey, Fukushima Medical University, Fukushima, Japan; ^3^Department of Public Health, Fukushima Medical University School of Medicine, Fukushima, Japan; ^4^Sendai City Mental Health and Welfare Center, Sendai, Japan; ^5^Faculty of Human Sciences, Musashino University, Tokyo, Japan

**Keywords:** community survey, depression, Fukushima nuclear disaster, mental health, psychological resilience

## Abstract

**Aim:** The aims of the present study were to develop and validate a psychological resilience scale reflecting the specificity of the Fukushima disaster, and to examine the effects of this scale on mental health.

**Methods:** The Fukushima Resilience Scale was developed based on data obtained from semi-structured interviews with seven people who had lived in the affected area of Fukushima Prefecture at the time of the disaster. The reliability and validity of the scale were then examined in cross-sectional studies conducted on 500 evacuees through an epidemiological mail survey. To examine the effects of the scale and disaster-related factors on the general mental health status of the respondents, a logistic regression analysis was performed using the six-item Kessler psychological distress scale.

**Results:** The newly developed scale consisted of a four-factor structure: “coping with stigma-related issues,” “sharing experiences of the disaster,” “action-oriented approach,” and “sense of support.” Internal consistency coefficients ranged from 0.66 to 0.79. The multivariable logistic regression analysis showed that the only significant association was between “action-oriented approach” (odds ratio = 1.26) and respondents with a K6 score <5 points.

**Conclusion:** The reliability and concurrent validity of the new developed scale in residents of the evacuation area of Fukushima Prefecture were acceptable. A significant association was found between “action-oriented approach” and good mental health among the evacuees, which suggests that this may improve resilience among people affected by the Fukushima disaster.

## Introduction

The Great East Japan Earthquake (GEJE) and the consequent severe accident at the Fukushima Daiichi Nuclear Power Plant (FDNPP) caused devastating damage to Fukushima Prefecture and its residents. Over 40,000 people remain evacuees inside or outside of Fukushima and continue to suffer from not only worry about the adverse health effects from exposure to radiation, but also unfounded rumors and public stigma (1). In fact, the result of a recent major survey conducted on evacuees by Fukushima Medical University indicated that as much as 7% of these evacuees are at risk of depression (2). In addition, more than 100 disaster-related suicides have been reported in Fukushima since the disaster, exceeding the numbers in Miyagi and Iwate Prefectures, which were affected mainly by the tsunami (3).

Regardless of such difficulties in terms of relief efforts, several items identified in panel data from Fukushima have suggested positive and hopeful change after the disaster (4). For example, remarkable increases in residential construction, agricultural production, job growth, and industrial output have been observed. However, although such physical improvements have been seen, few studies have revealed any positive changes in terms of psychological issues among disaster-affected people in Fukushima. In particular, psychological resilience factors, which should play an important role in recovery efforts after natural disasters (5), have yet to be comprehensively examined.

With regard to resilience, epidemiological studies in natural and technological disasters such as Nias–Simeulue earthquake (2005), Sichuan earthquake (2008), Haiti earthquake (2010), Hurricane Katrina (2005), and Deepwater Horizon Oil Spill accident (2010) showed substantial association between psychological problems and individual resilience (6–10). Studies performed in the Tohoku area also revealed that individual resilience among medical workers in the first year after the GEJE predicted high work engagement after 4 years (11), and that resilience among initial responders such as firefighters was associated with a low severity of post-traumatic stress disorder (PTSD) symptoms (12). In addition, one study involving high school students in Miyagi Prefecture showed an increase in those having high resilience, as well as a negative correlation between high resilience and depressive symptoms (13). Furthermore, a study of evacuees from a town located near the FDNPP suggested that resilience could be an important meditator of depression, PTSD, and other general mental health issues (14). Another recent study conducted on evacuees after the GEJE revealed a positive association between mental health recovery and desirable lifestyles and social networks, particularly in regard to social roles (15).

Although these studies suggested that psychological resilience could contribute to the retention or restoration of mental health among people affected by GEJE, in the case of complex disasters such as the Fukushima disaster that cause long-term psychological effects, resilience factors remain unclear. Especially in Fukushima, multidimensional psychosocial issues, including the fragmentation of communities and the weakening of social capital, resulting from situations of ambiguous loss and differing opinions toward radiation effects, exert long-term influence on the mental health of those affected (1). For example, in contrast with other prefectures affected mainly by tsunami (e.g., Miyagi and Iwate Prefectures), people experiencing the nuclear power plant accident in Fukushima Prefecture tended to relocate to remoter area across the country. Actually, there are over 40,000 still staying out of Fukushima Prefecture due to fear for radiation exposure or other related reasons; on the other hand, only a few evacuees who had met tsunami in Miyagi and Iwate Prefecture were evacuated outside their original prefectures. Such remote, prolonged evacuation of the Fukushima people, leading family separation and/or loss of their future plans as well as stable lives, could damage preexisting community structure and culture. In addition, the Fukushima evacuees have been more likely to be stigmatized due to stereotype and prejudice among the public relating to genetic effects of radiation exposure and/or compensation from Tokyo Electric Power Company. Such stigmatization is also considered to be one of very unique social consequences emerging among Fukushima evacuees, while not seen in natural disasters (1).

Given these specific features of Fukushima evacuees, resilience in relation to natural disasters may work in different ways. To clarify the situation regarding psychological resilience among disaster-affected people in Fukushima and establish further mental health care strategies, we attempted to identify resilience factors reflecting the specificity of the Fukushima disaster. For this purpose, a new scale to assess individual resilience among them should be made, because existing measurements such as the Connor–Davidson Resilience Scale (CD-RISC) focused on only psychological factors without including social factors (16). This study, thus, involved two procedures: the first (preliminary study) was to extract possible resilience factors relating to the Fukushima disaster through individual interviews with a small number of evacuees; the second (main study) was to conduct an epidemiological mail survey on a larger group of evacuees based on the above procedure. The findings obtained were expected to contribute to the establishment of mental health policies that promote recovery among those affected by the Fukushima disaster.

Incidentally, the concepts and definitions of resilience are considerably diverse (17).

In this study, we adopted the definition of resilience proposed by Rutter (18) in 1985, which was somewhat obscure but had been commonly used in domains of psychiatry and psychology; namely, resilience is *the ability to bounce back or cope successfully despite substantial adversity*.

## Preliminary Study (Study 1)

### Method

This preliminary study was conducted to develop a useful measurement for the main study (Study 2), as described later.

#### Participants

This study sample comprised seven affected people (1 man, 6 women; mean age ± standard deviation, 53.0 ± 13.6 years) who had once lived in the affected area of Fukushima Prefecture at the time of the disaster; five of these residents were thereafter evacuated to another area for at least 6 years. They all had experienced traumatic events related to the disaster, especially the FDNPP accident. For example, almost all of the evacuees felt extremely fearful and realized that they were driven to critical moments when hearing about the meltdown at the FDNPP. When this survey was conducted, all the participants were engaged in group activities supporting reconstruction in Fukushima, even though they were not professionals. People having apparent severe psychiatric symptoms such as depressive symptoms, including suicidal ideation, delusions, or other psychotic symptoms, were excluded. Written informed consents were obtained by mail from all the participants before their interviews. This survey was approved by the ethics review committee of Fukushima Medical University on July 29, 2016 (No. 2738).

#### Survey Procedure and Variables

All participants took part in individual semi-structured interviews with a psychiatrist (SN) or clinical psychologists (YT and MMo) at their workplace; each interview lasted about 60 min. After inquiring about their history and personal experience after the disaster, the participants were asked about matters relating to their resilience using the following questions: (1) Do you think something contributed to your life even after the disaster? If so, what?; (2) Do you think something changed in terms of your mind or lifestyle after the disaster? If so, what?; (3) Do you think you got something from the disaster? If so, what?; (4) Do you feel as though you have some pleasure or satisfaction now? If so, from what?; and (5) What do you think could contribute to happiness among disaster-affected people in the future?

These questions were considered necessary to assess whether there were some positive changes related to resilience among disaster-affected people, even after the experience of the disaster and subsequent long-term evacuation life.

#### Analysis

The results of the interviews were qualitatively analyzed using the KJ method with our research team (consisting of YT, SN, MMo, and AI). The KJ method was developed by Kawakita in the 1960s, and has been widely used in Japan for the sake of grouping data and making affinity diagrams (19).

## Results

As a result of the KJ method, items relating to resilience were classified into the following eight categories: (1) receiving support (e.g., support from family members or others); (2) positive acceptance in an evacuation area or other new address (e.g., good interaction in an evacuation area, participation in some social activities); (3) interaction with people evacuated from the same affected area (e.g., interaction with friends before the disaster occurred, opportunities to talk about life as an evacuee with each other); (4) work and related issues (e.g., employment or unemployment, job satisfaction, having a job that is somewhat helpful to others, good relationship with coworkers); (5) hobbies and pleasure; (6) changes in personal awareness (e.g., feeling of gratitude for daily life, self-efficacy, being able to do something new, acceptance of current difficulties); (7) sense of distance from one's hometown; and (8) reduction of anxiety related to radiation exposure.

These eight categories were composed of a total of 16 question items; these were arranged to devise the new resilience questionnaire for evacuees of the Fukushima disaster (Fukushima Resilience Scale; FRS) (Table 1).

**Table 1:** The items of the Fukushima Resilience Scale (FRS).

	**Items**	**Strongly disagree**	**Disagree**	**Neutral**	**Agree**	**Strongly agree**
1	Can you tell the residents of the area you evacuated to that you are evacuees?	1	2	3	4	5
2	Do you feel that you are accepted as evacuees to the residents of the area you evacuated to?	1	2	3	4	5
3	Do you feel that Fukushima citizens are accepted to people other than Fukushima Prefecture?	1	2	3	4	5
4	Have you been interacting with friends from the evacuation area?	1	2	3	4	5
5	Is there a place to meet with the residents of the area where you lived before evacuation?	1	2	3	4	5
6	Do you have places where you feel free to talk about the disaster and evacuation?	1	2	3	4	5
7	Have you ever been able to do new things in your life after the disaster?	1	2	3	4	5
8	Do you think that the disaster can not be helped, and there is no choice but to do what you can do?	1	2	3	4	5
9	Do you have time to enjoy yourself, such as hobbies?	1	2	3	4	5
10	Do you feel that you are helpful to others through your job(s), housework, or social activities?	1	2	3	4	5
11	Do you feel that you received support and encouragement on your daily life from your family/acquaintance after the disaster?	1	2	3	4	5
12	Do you feel that you received support and encouragement on your daily life other than your family/acquaintance after the disaster?	1	2	3	4	5

*Fukushima Resilience Scale (FRS) that were arranged to devise the new resilience questionnaire for evacuees of the Fukushima disaster, were finally composed of a total of 12 question items*.

### Main Study (Study 2)

#### Method

The main study was conducted using the new questionnaire devised in the preliminary study (Study 1).

#### Participants

This was a cross-sectional questionnaire survey of 1,000 residents of Fukushima Prefecture aged 20 years and above. Among these, 500 people from the evacuation area consisting of Tamura city, Minami-soma city, Kawamata town, Hirono town, Naraha town, Tomioka town, Okuma town, Futaba town, Namie town, Kawauchi village, Katsurao village, and Iitate village were selected. The Japanese government has indicated evacuation areas according to spatial radiation dose rates as follows: (1) difficult-to-return areas with a radiation dose rate ≥50 mSv per year; (2) residence-restricted areas with a radiation dose rate ≥20 and <50 mSv per year; and (3) areas where evacuation orders were ready to be lifted as of April 22, 2011. The residents of the evacuation area were forced to leave their homes at the direction of the Japanese government. In total, 500 people in the non-evacuation area were selected using a two-stage stratified random sampling method. We sent an anonymous, self-reporting postal questionnaire to participants from January to February 2018. In addition, 500 people were targeted from the evacuation area.

This survey was approved by the ethics review committee of Fukushima Medical University on October 10, 2017 (No. 29206). Part of the data obtained from the same participants was reported in another article (15) that analyzed and showed the recovery patterns of mental health among these individuals.

#### Survey Variables

**1) Disaster-related experiences**
Disaster-related experiences (e.g., housing damage, loss of family, relatives, or friends, separation from family members, and disaster-related loss of employment) were evaluated on a two-point scale, defined as “Experienced” or “Never.”
**2) Intention about future living place**
Intention about future living place was assessed on a four-point scale, defined as “Not yet determined,” “Returned,” “Intend to return,” and “Do not intend to return.”
**3) Economic status**
We assessed economic status using the following question “Do you feel that you can get by according to your current economic status?,” which was assessed on a five-point scale, defined as “Difficult,” “Somewhat difficult,” “Average,” “Somewhat adequate,” and “Adequate.” Occupational category was evaluated on a five-point scale, defined as “Employed,” “Owner,” “Part-time,” “Homemaker,” and “Unemployed.”
**4) Resilience**
We used the FRS devised in the preliminary study (Study 1). To assess its concurrent validity, we used the Connor–Davidson Resilience Scale (CD-RISC). The CD-RISC was developed to assess an individual's ability to cope with traumatic stress (16). It is composed of 25 items, all of which are rated on a five-point Likert scale, with higher scores indicating greater resilience. The reliability and validity of the Japanese version of the CD-RISC have been confirmed (20).
**5) General mental health status**
We used the 6-item Kessler psychological distress scale (K6), which is a self-administered measure used to screen for mood or anxiety disorders. Participants scoring 5–12 points were classified as having psychological distress (21), whereas those scoring 13–24 points were classified as having probable severe psychological distress (22). The reliability and validity of the Japanese version of the K6 have been confirmed (23).

#### Statistical Analysis

The factorial validity for the FRS for the triple disaster (earthquake, tsunami, and nuclear accident) was evaluated using an exploratory analysis utilizing the least-squares method with promax rotation. The number of factors was determined by minimum average partial (MAP) correlation and parallel analysis. We evaluated the internal consistency of the newly developed FRS using Cronbach's alpha. Concurrent validity was evaluated using a correlation analysis between the CD-RISC, FRS, and K6.

The chi-squared test was used to estimate the associations between general mental health status (K6 score) and disaster-related experiences, intention regarding future living place, and economic status. Next, a multivariable logistic regression model was carried out with general mental health status (K6 score) as the dependent variable. The independent factors used in this analysis were only those showing significant differences or tendencies according to the chi-squared test; these included economic status, disaster-related factors, and factors obtained from the above exploratory factor analysis. With regard to the independent factors, groups with a K6 score >5 in were adopted as a reference. All statistical analyses were performed using SPSS 25.0 (IBM Corp., Armonk, NY, USA).

## Results

### Participants

**Figure 1:**
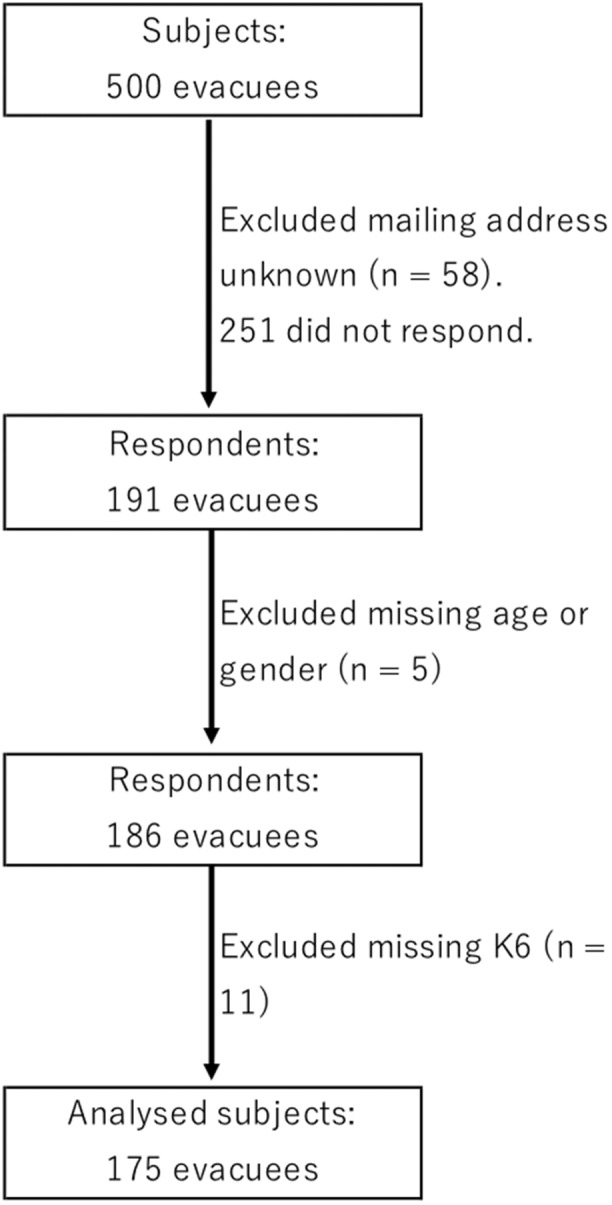
Flow chart of study procedures. From the targeted population (*n* = 500), 58 were excluded due to mailing address unknown. Two hundred fifty-one did not respond. Final number of valid responses was 175 after excluding respondents with missing data regarding age, gender or the 6-item Kessler psychological distress scale (K6).

After excluding 58 individuals that were returned to sender because no one was residing at the address, from among the remaining 442 questionnaires sent out, we received 191 responses (response rate, 43.2%). After excluding five respondents who failed to provide information regarding their age or gender, and 11 who did not complete the K6, the final study population consisted of 175 respondents (Figure 1).

### Respondent Characteristics

Finally, 175 respondents (82 men, 93 women; mean age ± standard deviation, 60.0 ± 14.2 year) were analyzed. In total, 69 respondents (37.1%) had a K6 score of ≤4 points, and 106 (62.9%) had a score of ≥5 points. In this study, the participants were divided into two groups for analysis (those with <5 points and those with ≥5 points). Table 2 shows the distribution of the participants' basic characteristics and disaster-related experiences. Difficult economic status and disaster-related loss of employment were significantly higher in the group with a K6 score of ≥5 points.

**Table 2:** Basic characteristics and disaster-related experiences of the participants.

	**Total (*****n*** **= 175)**		**K6 score <5 (*****n*** **= 69)**		**K6 score ≥5 (*****n*** **= 106)**		***P*-value**	***X*^**2**^**
	***n***	**%**	***n***	**%**	***n***	**%**		
**BASIC CHARACTERISTICS**								
**Gender**								
Male	82	100.0	37	45.1	45	54.9	0.15	2.09
Female	93	100.0	32	34.4	61	65.6		
**Age (y)**								
<40	20	100.0	11	55.0	9	450	0.23	2.94
40–64	79	100.0	32	40.5	47	59.5		
≥65	76	100.0	26	34.2	50	65.8		
**Occupation**								
Unemployed	71	100.0	22	31.0	49	69.0	0.41	4.20
Employed, owner, part-time, homemaker	101	100.0	47	46.5	54	53.5		
**Economic status**								
Difficult	48	100.0	11	22.9	37	77.1	0.01	7.55
Adequate/average	127	100.0	58	45.7	69	54.3		
**DISASTER-RELATED EXPERIENCE**								
**House damage**								
Experienced	74	100.0	26	35.1	48	64.9	0.32	0.99
Never	101	100.0	43	42.6	58	57.4		
**Loss of family, relatives, or friends**								
Experienced	51	100.0	15	29.4	36	70.6	0.08	3.02
Never	124	100.0	54	43.5	70	56.5		
**Separation from family members**								
Experienced	98	100.0	40	40.8	58	59.2	0.67	0.18
Never	77	100.0	29	37.7	48	62.3		
**Loss of employment**								
Experienced	63	100.0	18	28.6	45	71.4	0.03	4.86
Never	112	100.0	51	45.5	61	54.5		
**Intention about future living place**								
Not yet determined	24	100.0	7	29.2	17	70.8	0.27	1.20
Returned/intend to return/do not intend to return	149	100.0	61	40.9	88	59.1		

*This table shows the distribution of the participants' basic characteristics and disaster-related experiences. The chi-squared test showed that difficult economic status and disaster-related loss of employment were significantly higher in the group with a K6 score ≥5 points*.

### Factor Analysis

The MAP correlation indicated a one-factor solution, and parallel analysis indicated a five-factor solution. From the viewpoint of interpretability, we decided that the four-factor solution was appropriate. Namely, the exploratory factor analysis showed that the FRS consisted of a four-factor structure. Items with factor loadings <0.40 were deleted, and 12 items were extracted as follows: 1st factor, three items; 2nd factor, three items; 3rd factor, four items; and 4th factor, two items (Table 3). The overall Cronbach's alpha coefficient of the scale was 0.81. Cronbach's alpha coefficients for the four factors were 0.79, 0.77, 0.66, and 0.75, which accounted for 14.9, 23.0, 8.9, and 6.5% of the variance between items, respectively. Here, we named the 1st factor “coping with stigma-related issues,” the 2nd “sharing experiences of the disaster,” the 3rd “action-oriented approach,” and the 4th “sense of support.”

**Table 3:**
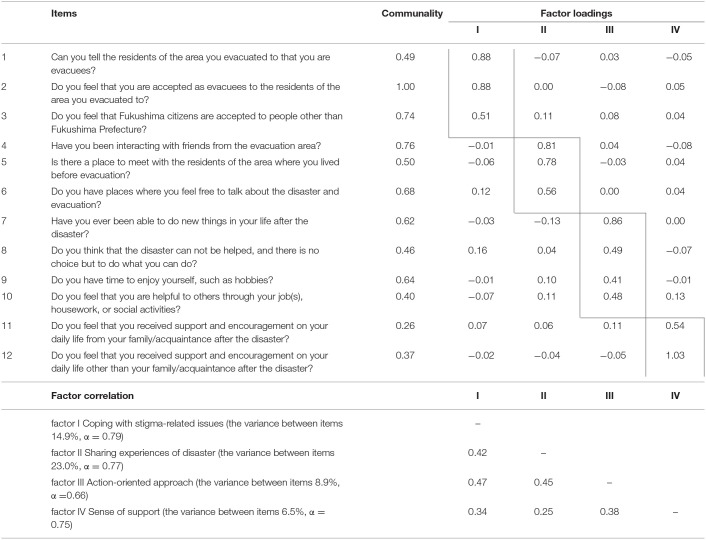
Results of exploratory factor analysis of the Fukushima Resilience Scale (FRS).

*The exploratory factor analysis showed that the FRS consisted of a four-factor structure and 12 items were extracted*.

The correlation analysis showed a significant correlation between the CD-RISC and total score of the FRS (*r* = 0.52, *p* < 0.001), as well as four factors: “coping with stigma-related issues” (*r* = 0.35, *p* < 0.001), “sharing experiences of the disaster” (*r* = 0.34, *p* < 0.001), “action-oriented approach” (*r* = 0.61, *p* < 0.001), and “sense of support” (*r* = 0.23, *p* < 0.01). Also, the correlation analysis showed a significant correlation between the K6 and total score of the FRS (*r* = −0.47, *p* < 0.001), as well as the CD-RISC (*r* = −0.54, *p* < 0.001).

### Multivariable Logistic Regression Model

In the multivariable logistic regression analysis, the independent factors were economic status, loss of family, relatives, or friends and loss of employment which were observed the association with a K6 score in the chi-squared test. The result showed that only “action-oriented approach” on the FRS (odds ratio = 1.26, 95% confidence interval: 1.09–1.45) was significantly associated with the group of respondents with a K6 score of <5 points (Table 4).

**Table 4:** Multivariable logistic regression analysis between K6 score and related factors.

	**OR**	**95%CI**		***p*-value**
		**Lower**	**Upper**	
**BASIC CHARACTERISTICS**				
**Economic status**				
Difficult	0.54	0.22	1.32	0.18
Enough/Average (Ref.)	1.00			
**DISASTER-RELATED EXPERIENCE**				
**Loss of family, relatives, or friends**				
Experienced	0.63	0.27	1.46	0.28
Never (Ref.)	1.00			
**Loss of employment**				
Experienced	0.63	0.29	1.35	0.23
Never (Ref.)	1.00			
**THE FUKUSHIMA RESILIENCE SCALE (FRS)**				
Coping with stigma-related issues	1.03	0.89	1.19	0.67
Sharing experiences of disaster	1.13	0.99	1.28	0.07
Action-oriented approach	1.26	1.09	1.45	0.002
Sense of support	0.87	0.73	1.04	0.12

*The multivariable logistic regression analysis showed that only “action-oriented approach” on the FRS was significantly associated with the group of respondents with a K6 score of <5 points*.

## Discussion

In the present study, we examined possible factors relating to the psychological resilience of people who were evacuated as a result of the accident at the FDNPP after the GEJE. First, semi-structured interviews were conducted with a few evacuees to clarify resilience-related factors and, as a result, a new questionnaire composed of 16 items was devised. Second, these new questionnaires were mailed to the target population of randomly selected individuals in the disaster area, and the results were analyzed mainly using exploratory factor analysis. The data gathered from the collected questionnaires (response rate: 43.2%) indicated high reliability (internal consistency) and concurrent validity with CD-RISC. The exploratory factor analysis also showed that the questionnaire was structured with four factors—“sense of support,” “action-oriented approach,” “coping with stigma-related issues,” and “sharing experiences of the disaster”—from which, 12 items were extracted (Table 3). The first two factors (“sense of support” and “action-oriented approach”) were considered to be similar to general, non-specific resilience factors demonstrated by the CD-RISC, whereas the second two factors (“coping with stigma-related issues” and “sharing experiences of the disaster”) may specifically relate to psychosocial issues caused by the Fukushima nuclear disaster and long-term evacuation life. As the result, while the CD-RISC focuses on only psychological factors that an individual might have, this newly developed scale named Fukushima Resilience Scale includes more social factors.

Previous studies have revealed that social support and fostering close relationships with other community members are important to enhance psychological resilience among disaster-affected people. Our results obtained from the factor analysis indicated the importance of good relationships with community members that had been established before the disaster, as well as good current relationships with those living in the towns that the evacuees relocate to. However, binomial logistic regression analysis including the above four factors showed that only one factor, “action-oriented approach,” was significantly associated with good mental health among the evacuees, as identified by the K6. The factor “action-oriented approach” was regarded as an important measure for resilient people (18), and represents several individual abilities related to making something to do on one's own, playing some social roles, and having leisure time. These results suggest the possibility that an “action-oriented approach” could contribute to the promotion of mental health among people affected by a major disaster. A previous study examining the influence of individual coping skills on psychological resilience also reported the significance of an action-oriented approach (24).

Many evacuees are experience ambiguous loss rather than apparent loss, such as the deaths of a loved one resulting from the tsunami (1), which may lead to the loss of certain social roles or individual life goals. Even in such ambiguous situations, evacuees who are able to set some positive new goals, do something meaningful such as recreation and foster good relationships with others, may also be able to maintain a healthy status. In the context of mental health care and treatment, promoting activities among evacuees through various types of interventions performed by different health care providers, including local health care centers, can be effective to reinforce resilience. In addition, if possible, behavioral activation used in cognitive behavioral therapy may contribute to the improvement of psychiatric symptoms such as depressive symptoms.

Despite not reaching significance level (*p* = 0.07), the factor “sharing experiences of the disaster,” which reflected the existence of good relationship with others even before the disaster, tended to be associated with good mental health. Given this factor, it may be effective to utilize positively social capital originally existing in different communities in Fukushima, even though such social capital has been facing a crisis, as described above. For example, numerous types of facilities or organizations, including non-profit organizations, offer community-based activities for evacuees. These resources have helped to ameliorate the fragmentation of communities in Fukushima caused by different reasons, such as long-term, repeated relocation, and differences in risk perception toward radiation exposure or delayed relief (1). Unfortunately, 8 years after the disaster, these resources remain inhibited by a lack of not only funds, but also human resources. Thus, national and municipal governments need to support these resources to develop and reestablish the strength of existing social capital.

On the other hand, several factors, including economic status, bereavement, and unemployment, were not associated with good mental health among the participants. These findings are inconsistent with previous studies conducted in regard to natural disasters. A recent meta-analysis of risk factors for depression after natural disasters revealed that serious loss experiences such as bereavement and unemployment were strongly associated with depression among affected people (25). One possible reason to explain this inconsistency is that, if we analyze the data using a group with more severe psychological distress (K6 score >13) as a reference, the loss experiences as described above might be associated with a healthy condition among the participants. Because of the difficulties involved with conducting a logistic regression analysis with a considerably small number of participants in the high distress group, we need to avoid adopting this model. Interestingly, a major mental health survey conducted on evacuees from Fukushima also showed that bereavement was not a significant factor influencing mental health, whereas radiation risk perception was the strongest factor (26). Considering this specific tendency in Fukushima, while an apparent loss experience such as bereavement was not a prominent factor affecting mental health status, many affected people in Fukushima might conceivably feel that the fragmentation or alternation of their communities was a more traumatic experience.

This study had several limitations. First, the study design was not longitudinal, but rather cross-sectional. The former can clarify more possible factors contributing to the resilience of affected people. Second, the target population of this study (Studies 1 and 2) was not large. In addition, we adopted the Rutter's concept as a definition of resilience in this study, whereas the concept of psychological resilience, essentially, is diverse and includes different ideas (27), For example, which level does resilience depend on individual or community? What does resilience stand for? Individual predispositions, including inheritance, some skills that can be acquired, or the course of recovery from disasters?

Despite these limitations, this study has shed light on resilience as opposed to vulnerability, which has been the focus of many previous studies. Furthermore, FRS newly developed in this study can be applied to other Chemical Biological Nuclear Explosive (CBRNE) disasters that might produce negative social reactions such as public stigma similar to the Fukushima disaster. Our results could help clarify the mental health care strategies that should be followed for people affected by technological disasters in public health care settings.

## Data Availability Statement

Requests to access the datasets should be directed to Yui Takebayashi, takeb-ky@fmu.ac.jp.

## Ethics Statement

The studies involving human participants were reviewed and approved by the ethics review committee of Fukushima Medical University. Written informed consent for participation was not required for this study in accordance with the national legislation and the institutional requirements.

## Author Contributions

HO, SY, MMa, and SN conceived and designed the framed study. SN, MMo, and YT conducted the interview in the preliminary study. SN, MMo, AI, and YT performed the analysis in the preliminary study. MMa, MO, SN, MMo, AI, SY, HO, and YT contributed to designing the questionnaire in the main study. MO conducted the questionnaire survey in the main study. YT analyzed the data in the main study. YT, HS, and MMa wrote the paper. All authors contributed to revisions of the manuscript and critical discussion.

## Conflict of Interest

The authors declare that the research was conducted in the absence of any commercial or financial relationships that could be construed as a potential conflict of interest.
